# Pneumocystis jirovecii Pneumonia in an HIV-Negative Patient Receiving Adjuvant Capecitabine and Oxaliplatin (CAPOX) Chemotherapy for Colon Adenocarcinoma: A Case Report

**DOI:** 10.7759/cureus.114640

**Published:** 2026-08-17

**Authors:** Clara Pinto, Margarida M Almeida, Alexandre C Lopes, João Faia, Ana S Martins

**Affiliations:** 1 Internal Medicine, Unidade Local de Saúde da Região de Aveiro, Aveiro, PRT

**Keywords:** capox chemotherapy, colon adenocarcinoma, immunosuppression, opportunistic infections, pneumocystis jirovecii

## Abstract

*Pneumocystis jirovecii* pneumonia (PJP) is a potentially life-threatening opportunistic infection, classically associated with human immunodeficiency virus (HIV) infection but increasingly recognized in non-HIV immunocompromised hosts, including patients with solid tumors undergoing chemotherapy. We present the case of a 57-year-old woman with stage II (pT3N0M0) mucinous colon adenocarcinoma who developed PJP following adjuvant capecitabine and oxaliplatin (CAPOX) chemotherapy, without exposure to prolonged corticosteroids or severe cytopenia. The patient presented with progressive exertional dyspnea and a productive hemoptoic cough the week after the last chemotherapy cycle. Laboratory tests revealed mild lymphopenia with preserved neutrophil counts and elevated lactate dehydrogenase (LDH). Chest computed tomography demonstrated bilateral ground-glass opacities, and bronchoalveolar lavage (BAL) confirmed the presence of *P. jirovecii *by immunofluorescence. Treatment was initiated with intravenous trimethoprim-sulfamethoxazole for seven days, followed by two weeks of oral therapy, without adjunctive corticosteroid therapy. The patient made a full clinical recovery.

This case highlights the need to consider PJP in non-HIV immunosuppressed patients, particularly those with solid tumors receiving chemotherapy, even in the absence of classical risk factors such as severe cytopenia or prolonged corticosteroid use. Early diagnostic suspicion and targeted microbiological testing are essential, given the rapid progression and high mortality associated with PJP in this population.

## Introduction

*Pneumocystis jirovecii* pneumonia (PJP) is a potentially life-threatening opportunistic infection caused by the fungus *P. jirovecii*. Although usually associated with human immunodeficiency virus (HIV)-positive patients, PJP is increasingly recognized among non-HIV immunocompromised patients, including those receiving chemotherapy, corticosteroids, or other immunosuppressive agents [1,2]. PJP is a serious opportunistic infection typically linked to HIV infection, but the majority of cases now occur in non-HIV immunocompromised patients, particularly those with malignancies or receiving immunosuppressive therapies [2]. In patients with solid tumors, PJP remains relatively rare compared to hematologic malignancies, yet it carries a considerable risk of morbidity and mortality, sometimes exceeding that observed in HIV-positive populations [2-4].

The pathogenesis of PJP in non-HIV hosts is multifactorial, involving impairments of cell-mediated immunity due to chemotherapy, corticosteroid exposure, lymphocytopenia, or radiation therapy. Some chemotherapy regimens used for solid tumors, such as capecitabine and oxaliplatin (CAPOX), can transiently suppress cellular immunity, predisposing patients to opportunistic infections even in the absence of corticosteroid exposure [5].

Early recognition and prompt treatment are essential, as clinical and radiologic findings often overlap with bacterial or viral pneumonias, and delayed diagnosis carries a high risk of significant morbidity and mortality [2,6,7]. We report a case of PJP occurring in an HIV-negative 57-year-old woman during adjuvant chemotherapy for colon adenocarcinoma, highlighting the diagnostic and management challenges encountered in this setting.

## Case presentation

History and physical examination

A 57-year-old woman with a history of stage II (pT3N0M0) mucinous adenocarcinoma of the descending colon underwent left hemicolectomy approximately five months prior to admission, followed by adjuvant chemotherapy with CAPOX. She completed four cycles, with the last session 12 days before admission, and had no history of corticosteroid exposure, specifically including no exposure to dexamethasone or other steroids as part of her antiemetic prophylaxis regimen. Past medical history included chronic heart failure (New York Heart Association (NYHA) class III), hypertensive heart disease with asymmetric septal hypertrophy and preserved left ventricular ejection fraction (56%), atrial fibrillation anticoagulated with apixaban, hypertension, type 2 diabetes mellitus, and multinodular goiter with euthyroidism. There was no history of chronic lung disease.

Nine days before admission (three days after her last chemotherapy session), the patient developed progressive fatigue and myalgias, which she initially attributed to the chemotherapy. Two days before admission, she was evaluated in the emergency department (ED) for similar complaints and was medicated with furosemide 20 mg daily. On the day of admission, she returned to the ED with persistent exertional dyspnea, orthopnea, intermittent chest discomfort, and new-onset productive cough with mild hemoptysis, without fever. Physical examination revealed stable hemodynamics and oxygen saturation of 98% on room air. Chest auscultation disclosed bilateral crackles and intermittent wheezing. Before the chemotherapy session, the patient did not have cytopenias.

Diagnostic assessment

**Figure 1:**
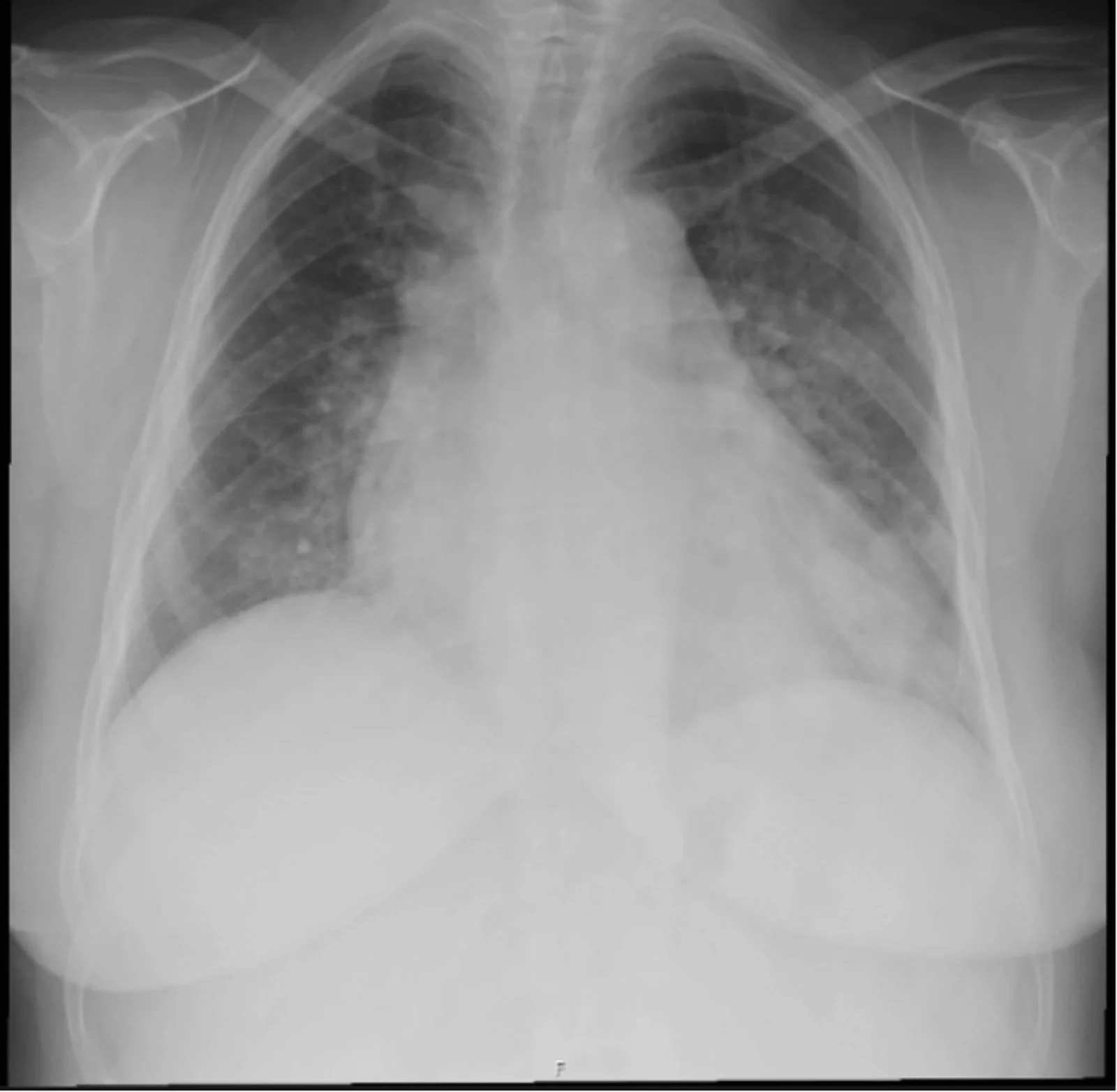
Chest X-ray performed on admission, showing no evident consolidations.

Following the initial clinical assessment, a comprehensive diagnostic workup was initiated. Initial laboratory results at admission showed hemoglobin of 12.2 g/dL, platelets of 104 × 10⁹/L, international normalized ratio (INR) of 1.4, lymphocytes of 1.07 × 10⁹/L, and a negative D-dimer. Lactate dehydrogenase (LDH) was 323 U/L, C-reactive protein (CRP) 3.49 mg/dL, and procalcitonin 0.03 ng/mL. Arterial blood gas on room air revealed pH of 7.43, pO₂ of 90.6 mmHg, pCO₂ of 34.3 mmHg, HCO₃ of 22.2 mmol/L, and lactate of 4.1 mmol/L (later normalized). A chest radiograph (Figure 1) showed new bilateral diffuse perihilar opacities compared to prior imaging three days before, without signs of congestion.

**Figure 2:**
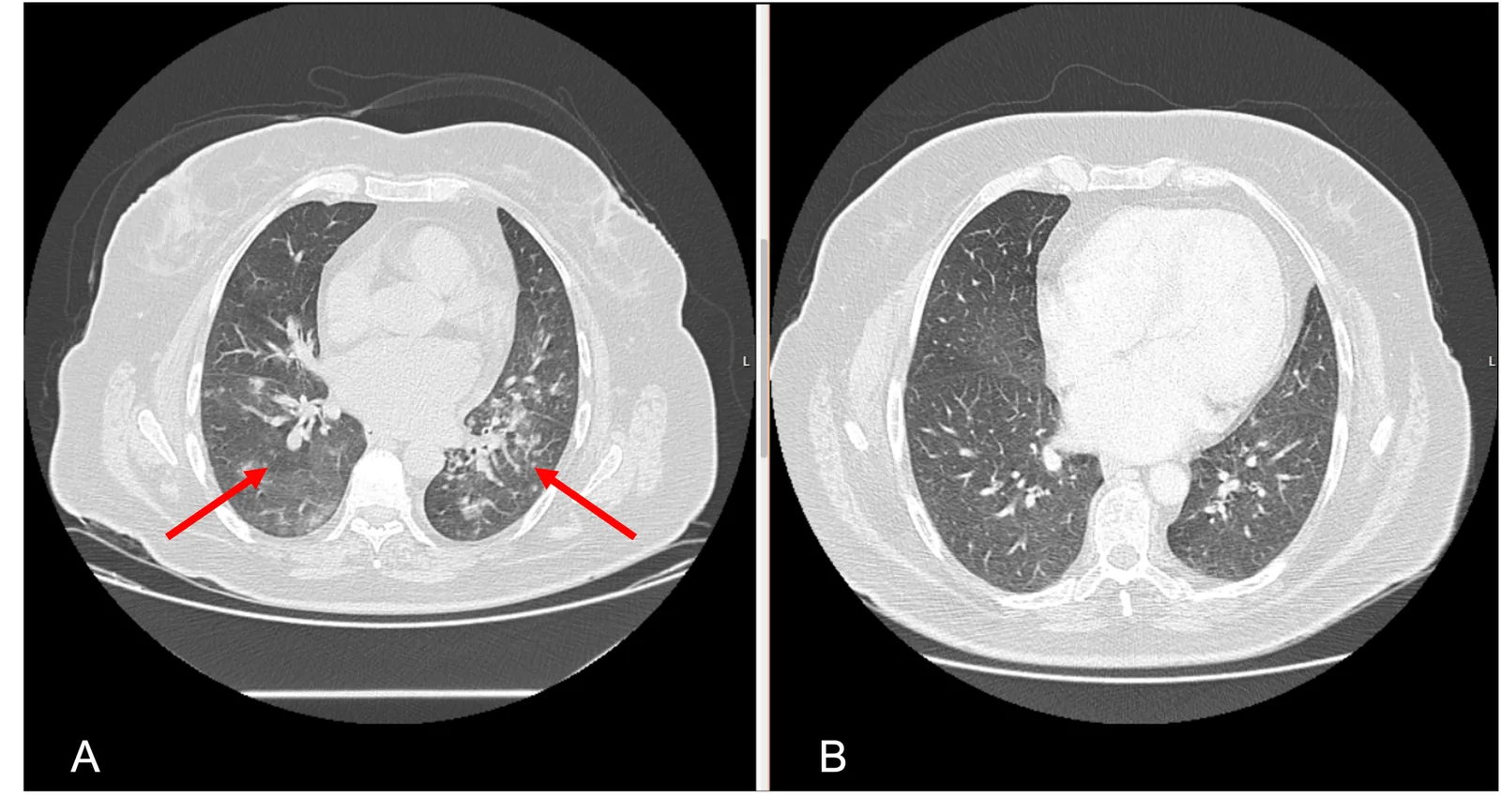
Chest CT findings at admission and follow-up. (A) Axial CT slice at admission showing bilateral ground-glass opacities (arrows), predominantly perihilar. (B) Follow-up CT after targeted treatment, showing complete resolution of the previous findings. CT: computed tomography

Computed tomography (CT) angiography of the chest excluded pulmonary embolism and revealed multiple bilateral ground-glass opacities, predominantly perihilar, mainly in the lower lobes and left upper lobe, suggesting an inflammatory process (Figure 2A). A respiratory viral polymerase chain reaction (PCR) panel (SARS-CoV-2, influenza A/B, respiratory syncytial virus, and adenovirus), *Mycoplasma pneumoniae*, and *Legionella pneumophila* tests were negative, as were blood cultures. The patient was evaluated by Cardiology in the ED, which considered the process non-cardiogenic and excluded acute heart failure. After discussion with Pulmonology, empirical intravenous amoxicillin/clavulanate and azithromycin were started for presumed atypical pneumonia in an immunocompromised patient.

A bronchoscopy with bronchoalveolar lavage (BAL) was performed as the pulmonary opacities persisted without a clear etiology. BAL cytology showed total cellularity of 500 cells/mm³, with 25% lymphocytes and an inverted CD4/CD8 T-cell ratio of 0.23 (CD4+ helper T-cells 18%, CD8+ cytotoxic T-cells 78%) (Table 1). Although the sample was collected on Day 8, a delay in laboratory reporting postponed confirmation of the immunofluorescence result until Day 28, at which point targeted therapy was initiated. Indirect immunofluorescence was positive for *P. jirovecii* (20 oocysts observed). Cytomegalovirus PCR and HIV serology were negative. Peripheral lymphocyte immunophenotyping revealed total lymphocytes at 1.04 × 10⁹/L, CD4 at 518/mm³, CD8 at 188/mm³, and an absence of CD19+ B-cells (Table 2).

**Table 1:** Cytological analysis and microbiological findings from BAL, showing lymphocytic predominance and an inverted CD4/CD8 ratio. Bold values indicate results that fall outside the reference range listed in the "Reference range" column - that is, clinically abnormal findings. BAL: bronchoalveolar lavage; CD4: cluster of differentiation 4; CD8: cluster of differentiation 8

Parameter	Result	Reference range
Total cells	500/mm^3^	<200/mm^3^
Macrophages	42%	>80%
Lymphocytes	25%	<15%
Neutrophils	15%	<3%
CD4	18%	-
CD8	78%	-
CD4/CD8 ratio	0.23	1.0-1.5
*Pneumocystis jirovecii* detection	Positive	Negative

**Table 2:** Peripheral lymphocyte immunophenotyping results, showing T-cell depletion and absence of B-cells. Bold values indicate results that fall outside the reference range listed in the "Reference range" column - that is, clinically abnormal findings. CD4: cluster of differentiation 4; CD8: cluster of differentiation 8; CD19: cluster of differentiation 19

Parameter	Result	Reference range
Lymphocytes	1.04 × 10⁹/L	1.50-4.00
CD4+ T-cells	518/mm^3^	700-1,100
CD8+ T-cells	188/mm^3^	500-900
CD19+ B-cells	Absent	100-500

Therapeutic intervention

The patient started targeted therapy with intravenous trimethoprim-sulfamethoxazole (TMP-SMX) 800/160 mg every 12 hours for seven days, followed by oral therapy to complete a 21-day course. Given the absence of hypoxemia (oxygen saturation 98% on room air; PaO₂ 90.6 mmHg; alveolar-arterial gradient approximately 16 mmHg, within the expected range for age), corticosteroids were not used. She tolerated the treatment and showed gradual clinical improvement with a better functional capacity throughout hospitalization. The laboratory findings are summarized in Table 3.

**Table 3:** Longitudinal laboratory data: comparison between pre-chemotherapy baseline, admission (with PJP symptoms), and post-treatment recovery. Bold values indicate results that fall outside the reference range listed in the "Reference range" column - that is, clinically abnormal findings. -: parameter not measured; ED: emergency department; LDH: lactate dehydrogenase; CRP: C-reactive protein; NT-proBNP: N-terminal pro-B-type natriuretic peptide; PJP: *Pneumocystis jirovecii* pneumonia

Parameter	Baseline (pre-chemotherapy)	Admission in ED	Post-treatment (Day 59)	Reference range
Hemoglobin	14.3 g/dL	12.2 g/dL	13.7 g/dL	11.5-16.5
Neutrophils	2.85 x 10⁹/L	2.57 x 10⁹/L	2.81 x 10⁹/L	2.00-7.50
Platelets	187 x 10⁹/L	104 x 10⁹/L	179 x 10⁹/L	150-500
Lymphocytes	1.56 x 10⁹/L	1.07 x 10⁹/L	1.09 x 10⁹/L	1.50-4.00
LDH	242 U/L	323 U/L	178 U/L	100-190
CRP	-	3.49 mg/dL	<0.05 mg/dL	0.00-0.50
Procalcitonin	-	0.03 ng/mL	0.01 ng/mL	<0.10
NT-proBNP	-	2,993 pg/mL	135 pg/mL	0-125

Follow-up and outcome

At follow-up (Day 59), a pulmonary CT demonstrated complete resolution of the previously described ground-glass opacities (Figure 2B), and most laboratory values returned to baseline levels, although mild lymphopenia (1.09 × 10⁹/L) persisted, showing minimal improvement compared to admission - a pattern consistent with the delayed lymphocyte recovery previously described after cytotoxic chemotherapy [5]. Due to the severity of the opportunistic infection and the associated immune vulnerability, chemotherapy was subsequently discontinued. Despite this residual lymphopenia, no secondary prophylaxis was prescribed; the patient remained closely monitored, was asymptomatic, and showed no signs of recurrence during follow-up. The temporal sequence of the clinical case, from chemotherapy initiation to therapeutic resolution, is summarized in Table 4.

**Table 4:** Clinical timeline: sequence of events from chemotherapy initiation to diagnostic procedures and therapeutic resolution, expressed relative to the day of hospital admission (Day 0). CAPOX: capecitabine and oxaliplatin; ED: emergency department; CT: computed tomography; BAL: bronchoalveolar lavage; IV: intravenous

Timeline (relative to admission)	Event
Month -3	Initiation of the first cycle of CAPOX chemotherapy.
Day -12	Completion of the fourth and final cycle of CAPOX.
Day -9	Onset of progressive fatigue and myalgias.
Day -2	First ED visit; symptomatic treatment with furosemide prescribed.
Day 0 (admission)	Second ED visit: exertional dyspnea, chest pain, hemoptoic cough.
Day 1	Pulmonary CT reveals bilateral ground-glass opacities.
Day 8	Bronchoscopy with BAL performed.
Day 28	*Pneumocystis jirovecii* immunofluorescence result reported; initiation of targeted IV trimethoprim-sulfamethoxazole.
Day 39	Completion of IV therapy and transition to oral treatment.
Day 59	Follow-up CT confirms resolution of pulmonary infiltrates.

## Discussion

This case illustrates a rare but serious presentation of PJP in a non-HIV patient following CAPOX chemotherapy, in the absence of traditional risk factors such as corticosteroid exposure or severe neutropenia. Although PJP is most commonly associated with HIV infection and profound immunosuppression, its occurrence in patients with solid tumors receiving cytotoxic chemotherapy is increasingly recognized [1,2]. This observation has been supported by multiple retrospective studies that highlight chemotherapy-induced transient immune dysfunction as a risk factor for PJP in non-HIV hosts [2,8].

Several factors contributed to transient immunosuppression in this patient. Cumulative cytotoxic exposure from CAPOX therapy, mild lymphopenia (1.07 × 10⁹/L), and selective depletion of B-cells and CD4+/CD8+ T-cell subsets created a permissive environment for opportunistic infection [5,8]. Peripheral immunophenotyping confirmed significant T-cell depletion, particularly CD4+ cells (518/mm³), and marked absence of CD19+ B-cells, consistent with moderate immunosuppression (Table 2). BAL cytology demonstrated lymphocytic predominance with an inverted CD4/CD8 ratio (0.23), further supporting an opportunistic etiology (Table 1) [1,7]. This T-cell depletion likely serves as the central mechanism increasing the risk for PJP in this setting, drawing a direct parallel with the immunological vulnerability observed in HIV infection [1,5].

The preserved marrow morphology and selective lymphocyte depletion suggest a direct cytotoxic or immunomodulatory mechanism of chemotherapy, rather than marrow infiltration or autoimmune destruction. Similar immune alterations have been described with oxaliplatin-containing regimens, where increased T-cell apoptosis and impaired thymic regeneration contribute to delayed immune recovery [5]. Taken together, these findings suggest that CD4+ T-cell depletion is the central mechanism underlying PJP susceptibility in this patient, mirroring the immunological vulnerability classically described in HIV-associated disease.

Clinically, PJP in non-HIV oncology patients carries high morbidity and mortality due to delayed recognition [2,3]. Typical imaging patterns include bilateral, symmetric ground-glass opacities with perihilar or diffuse distribution (Figure 2) [6,9], and, as in this case, low procalcitonin and absence of bacterial pathogens should raise suspicion for atypical infection [3]. BAL with immunofluorescence remains the diagnostic gold standard [1,7]. The mainstay of therapy is high-dose TMP-SMX for 21 days [1,3]. Adjunctive corticosteroids are reserved for moderate-to-severe hypoxemia, which was absent in this patient [10]. Despite a delay in treatment initiation due to laboratory reporting, prompt clinical recognition and targeted therapy were ultimately associated with favorable outcomes, and the patient responded fully without corticosteroid use. Although clinical suspicion prompted timely bronchoscopy by Day 8, a subsequent delay in laboratory reporting postponed treatment initiation until Day 28 - a limitation that underscores the importance of expedited communication pathways between laboratory and clinical teams once a high-risk diagnosis is suspected.

This case underscores the importance of considering PJP in solid tumor patients presenting with subacute respiratory symptoms and bilateral interstitial infiltrates, even in the absence of classical risk factors such as corticosteroid use or profound cytopenia [1,2]. Awareness of chemotherapy-induced lymphopenia as a risk factor, along with integrated immune monitoring, may facilitate early diagnosis and improve outcomes [5,8]. In selected patients with prolonged lymphopenia (<500 CD4+/mm³) or combination immunosuppression, prophylactic TMP-SMX may be justified to prevent opportunistic infections [3].

## Conclusions

PJP can occur in HIV-negative patients undergoing adjuvant chemotherapy for solid tumors, even in the absence of traditional risk factors such as corticosteroid exposure or severe cytopenia. Recognition of subtle chemotherapy-induced immunologic dysfunction and prompt diagnostic testing via BAL are crucial to avoid delayed diagnosis and improve clinical outcomes, given the high mortality associated with PJP in non-HIV hosts. This case underscores the importance of maintaining a high clinical suspicion for opportunistic infections in oncology patients presenting with interstitial infiltrates, regardless of their classic risk profile.
